# Socioeconomic status and lifestyle patterns in the most common cancer types-community-based research

**DOI:** 10.1186/s12889-023-16677-6

**Published:** 2023-09-05

**Authors:** Niclas Sandström, Mikael Johansson, Antti Jekunen, Heidi Andersén

**Affiliations:** 1 Cancer Clinic, Vaasa Central Hospital, Sandviksgatan 2-4, 65130 Vaasa, Finland; 2 Department of Radiation Sciences, Oncology, Umeå University, Umeå, Sweden; 3 Faculty of Medicine, University of Turku, Turku, Finland; 4 Faculty of Medicine and Health Technology, Tampere University, Tampere, Finland

**Keywords:** Socioeconomic status, Lifestyle, Health equity, Health literacy, Cancer

## Abstract

**Introduction:**

As the global burden of chronic cancer increases, its correlation to lifestyle, socioeconomic status (SES) and health equity becomes more important. The aim of the present study was to provide a snapshot of the socioeconomic and lifestyle patterns for different cancer types in patients at a Nordic tertiary cancer clinic.

**Materials and methods:**

In a descriptive observational study, questionnaires addressed highest-attained educational level, occupational level, economy, relationship status, exposures, and lifestyle habits. The questionnaire was distributed to all cancer patients attending the cancer clinic. Treating physicians added further information about the cancer disease, including primary origin, pathology report, TNM-classification and stage.

**Results:**

Patients with lung cancer had the lowest SES, and patients with gastrointestinal (GI) cancer, other cancer types and prostate cancer had the second, third and fourth lowest SES, respectively. However, breast cancer patients had the highest SES. Lifestyle and exposure patterns differed among the major cancer types. Lung cancer patients reported the highest proportion of unfavourable lifestyle and exposure patterns, and patients with GI cancer, prostate cancer and other cancer types had the second, third and fourth highest proportion of unfavourable lifestyle and exposure patterns, respectively. The most favourable exposure and lifestyle patterns were observed in breast cancer patients.

**Conclusions:**

The present study indicated significant socioeconomic and lifestyle differences among cancer types at a Nordic cancer centre, with differences in lifestyle being more prominent than socioeconomic differences.

## Introduction

The global burden of cancer is increasing and at least in high-income countries the proportion of cancer in elderly adults has increased [1, 2]. Cancer incidence is affected by lifestyle [3]. Lifestyle in turn is affected by socioeconomic status (SES) that further determinates living conditions, nutrition, physical activity, smoking habits, and exposures at work [4–7]. Our hypothesis was that socioeconomic and lifestyle factors needing attention differ between cancer types.

Several studies have shown that low SES is related to an unfavourable outcome based on cancer characteristics, cancer stage and cancer treatment regardless of cancer type and gender. For example, women with breast cancer and lower SES receive less sentinel lymph node biopsies and radiotherapy after surgery compared to women with higher SES. Moreover, women with lower SES have a two-fold risk of late-stage breast cancer at diagnosis regardless of cancer characteristics and detection mode (screening vs. clinical signs) [8, 9]. In men, low SES is associated with high-risk prostate cancer, longer waiting times for radical prostatectomy or radiotherapy and primary advanced disease [10]. For colorectal cancer, lower SES is associated with a worse outcome in patients with stage I-III cancer undergoing curative surgery. Low SES is also associated with an increased rate of postoperative complications [11]. This disparity in cancer-health may be alleviated by identifying vulnerable patient groups.

The present community-based study aimed to investigate whether the socioeconomic and lifestyle patterns differ among major cancer types, at a Nordic tertiary cancer centre. There are documented health differences between Swedish and Finnish speaking natives, however the disparities in cancer care remains unexplored [12–14]. This is particularly important in a Nordic country with assumed equal access to cancer care. Vulnerable patients may benefit from additional resources to reduce health disparities.

## Materials and methods

### Study design and participants

A descriptive study between December 20, 2021 and March 18, 2022 in Vaasa Central Hospital Cancer Clinic. During this time, 8559 contacts including telephone calls and 710 new referrals to the clinic were registered. A questionnaire consisting of 21 questions adapting wording from previous research [15–20] was selected focusing on factors important at baseline in oncology unit.

**Fig. 1:**
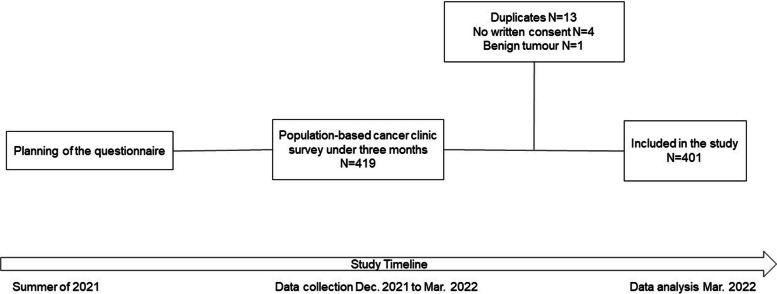
Flowchart of the study

The participants all gave written informed consent. The questionnaire was not distributed to patients in terminal stage due to ethical reasons. Participation in the study did not affect the care of the patients. The patients were asked to report the patient-related factors as they were during completion of the survey. After consent was given and the patient completed the survey, the treating physicians added further details about the cancer, including primary origin of the tumour, pathology report, TNM staging and cancer stage. When multiple cancers were identified in a patient, the cancer that was in active treatment or the reason for the visit was considered the primary tumour and staged accordingly. The questionnaires were mostly on paper and transferred to Research Electronic Data Capture (REDCap; Vanderbilt University, TN, USA) for digital processing. Figure 1 shows the flowchart of the study.

### Statistical analyses

Analyses were conducted using IBM SPSS Statistics version 28.0 (IBM Corp, NY, USA). Statistical significance was defined as a two-tailed *P* < 0.05 with a 95% confidence interval. The demographical characteristics and quartiles of the study population were analysed using frequencies. The socioeconomic and lifestyle patterns among cancer types were assessed using cross-tabulation with Chi-square analysis. Statistical significance of medians was determined using nonparametric tests with the Kruskal–Wallis test.

## Results

### Patient characteristics

In total, the present study included 401 participants with a median age of 70.0 years (Table 1). The response rate was 56.5% (710 referrals). In the population, there were more men, and the primary language was Finnish. The major cancer groups identified in the present study were breast cancer, lung cancer, gastrointestinal (GI) cancer, prostate cancer and other cancer types. The group defined as other cancer types consisted of pancreatic cancer (*n* = 16), glioma/glioblastoma (*n* = 13), malignant melanoma (*n* = 13), gynaecological malignancy (*n* = 12), head/neck carcinoma (*n* = 9), renal carcinoma (*n* = 9), neuroendocrine tumour (*n* = 8), urothelial carcinoma (n = 7), sarcoma (*n* = 6), haematologic malignancy (*n* = 6), testicular cancer (*n* = 5), thyroid cancer and mesothelioma in smaller proportions. The most common cancer stage of the study population was late cancer stage (i.e., WHO stage IV; 49.0%).

**Table 1:** Demographics and comparison of socioeconomic factors among cancer types according to Chi-square analysis with Pearson correlation

	**All**	**Breast Cancer**	**Lung Cancer**	**GI cancer**	**Prostate Cancer**	**Other Cancers**	***p*-value**
	N (%)	N (%)	N (%)	N (%)	N (%)	N (%)	
**Number**	401 (100%)	89 (22.2%)	67 (16.7%)	69 (17.2%)	69 (17.2%)	107 (26.7%)	
**Sex**							*P* < 0.001
Male	208 (51.9%)	0 (0%)	44 (65.7%)	40 (58.0%)	69 (100%)	55 (51.4%)	
Female	193 (48.1%)	89 (100%)	23 (34.3%)	29 (42.0%)	0 (0%)	52 (48.6%)	
**Age**							P < 0.001
Median (Q1, Q3)	70 (61, 76)	63.0 (50, 72)	72.0 (68, 76)	70.0 (63.5, 75)	74 (69, 77)	68 (59, 76)	
**Stage**							*P* < 0.001
I	50 (12.5%)	27 (30.3%)	2 (3.0%)	2 (2.9%)	7 (10.1%)	12 (11.2%)	
II	64 (16.0%)	24 (26.9%)	7 (10.4%)	7 (10.1%)	13 (18.8%)	13 (12.1%)	
III	83 (20.7%)	21 (23.6%)	13 (19.4%)	20 (29.0%)	11 (15.9%)	18 (16.8%)	
IV	189 (49.0%)	17 (19.1%)	45 (67.2%)	40 (58.0%)	38 (55.1%)	49 (45.8%)	
**Primary Language**							*P* < 0.001
Finnish	240 (59.9%)	58 (65.2%)	55 (82.1%)	37 (53.6%)	36 (52.2%)	54 (50.5%)	
Swedish	161 (40.1%)	31 (34.8%)	12 (17.9%)	32 (46.4%)	33 (47.5%)	53 (49.5%)	
**Education**							*P* = 0.015
Compulsory	122 (30.4%)	19 (21.3%)	27 (40.3%)	21 (30.4%)	26 (37.7%)	29 (27.1%)	
Upper Secondary	152 (37.9%)	28 (31.5%)	27 (40.3%)	26 (37.7%)	24 (34.8%)	47 (43.9%)	
Tertiary	127 (31.7%)	42 (47.2%)	13 (19.4%)	22 (31.9%)	19 (27.5%)	31 (29.0%)	
**ISCO-08**							*P* = 0.175
Skill level 1–2	253 (63.1%)	48 (54.0%)	48 (71.6%)	46 (66.7%)	41 (59.4%)	70 (65.4%)	
Skill level 3–4	148 (36.9%)	41 (46.0%)	19 (28.4%)	23 (33.3%)	28 (40.6%)	37 (34.6%)	
**Can afford a sudden payment of €1200**							*P* = 0.597
Yes	260 (64.8%)	55 (61.8%)	42 (62.7%)	42 (60.9%)	49 (71.0%)	72 (67.3%)	
No	131 (32.7%)	32 (38.2%)	24 (37.3%)	25 (39.1%)	18 (29.0%)	32 (32.7%)	
**Income per month**							*P* = 0.574
High- or medium-income category	282 (70.3%)	65 (73.0%)	44 (65.7%)	46 (66.7%)	52 (75.4%)	75 (70.1%)	
Low-income category	103 (25.7%)	18 (27.0%)	21 (34.3%)	20 (33.3%)	16 (24.6%)	28 (29.9%)	
**Relationship status**							*P* = 0.357
Living with someone	284 (70.8%)	62 (69.7%)	45 (67.2%)	48 (69.6%)	55 (79.7%)	74 (69.2%)	
Living alone	112 (27.9%)	27 (30.3%)	21 (32.8%)	20 (30.4%)	12 (20.3%)	32 (30.8%)	
**Health Literacy self-reported 0–100 (n = 377)**							*P* = 0.006
Median (Q1,Q3)	53 (51, 56)	62 (56, 65)	45 (41, 54)	45 (40, 54)	54 (51, 64)	51 (47, 58)	

*GI* Gastrointestinal, *Q1* Lower quartile, *Q3* Upper quartile, *ISCO-08* International standard classification of occupations

### Socioeconomic status

Compulsory education was reported as the highest attained educational level with a percentage of 30.4%. The dominant occupational group was ISCO-08 occupational skill levels one and two combined. Regarding economy, 25.7% of the participants did not have an income higher than €1200 per month, and 32.7% of the participants could not afford a sudden payment of €1200. The majority of participants lived with someone, and 27.9% of the participants reported living alone. Participants were interviewed of their perception of their health literacy with scale 0–100, low to high. There was a significant trend of lower perception among lung cancer and GI cancer participants than breast cancer participants (Table 1). When assessing the distribution of risk factors by age groups, those over 70 years old had lower education but a higher occupational level when compared to participants under 70 years old (Table 2).

**Table 2:** Comparison of cancer patients < 70 years and ≥ 70 years according to Chi-square analysis with Pearson correlation

**Patient-related variables**	** < 70 years**		** ≥ 70 years**		***p*-value**
	N	%	N	%	
**Education**					***p* < 0.001**
Compulsory	25	12.9%	97	46.9%	
Upper secondary	83	42.8%	69	33.3%	
Tertiary	86	44.3%	41	19.8%	
**Occupational level**					***p* < 0.001**
1–2	104	53.6%	58	28.0%	
3–4	90	46.4%	149	72.0%	
**Can afford a sudden payment of € 1200**					*p* = 0.252
No	69	36.3%	62	30.8%	
Yes	121	63.7%	139	69.2%	
**Income per month**					*p* = 0.685
Under € 1200	48	25.8%	55	27.6%	
Over € 1200	138	74.2%	144	72.4%	
**Living with someone**					*p* = 0.369
No	50	26.2%	62	30.2%	
Yes	141	73.8%	143	69.8%	
**Smoking status**					*p* = 0.735
Current smoker	19	9.8%	17	8.4%	
Ex-smoker	89	45.9%	88	43.6%	
Never smoker	86	44.3%	97	48.0%	
**Exposure to second-hand smoke**					*p* = 0.194
Yes	38	19.9%	52	25.4%	
No	153	80.1%	153	74.6%	
**Exposure to VGDF**					*p* = 0.075
Yes	60	31.6%	82	40.2%	
No	130	68.4%	122	59.8%	
**Exposure to asbestos**					*p* = 0.086
Yes	19	10.3%	32	16.2%	
No	166	89.7%	165	83.8%	
**Alcohol habits**					*p* = 0.054
Consumes alcohol	113	60.8%	102	51.0%	
Does not consume	73	39.2%	98	49.0%	
**Daily portions of greens in diet**					*p* = 0.126
1–3	119	64.3%	139	71.6%	
4–6	66	35.7%	55	28.4%	
**Activity per day in hours**					*p* = 0.397
1–2	94	51.6%	107	56.0%	
3–6	88	48.4%	84	44.0%	
**Exercise according to recommendations**					*p* = 0.052
No	95	51.1%	120	60.9%	
Yes	91	48.9%	77	39.1%	

Occupational level as in ISCO-08, International Standard Classification of Occupations. *VGDF* Vapours, gas, dust and fumes. Chi-square analysis with Pearson correlation

### Lifestyle

The majority of participants reported a smoking history (Table 3). Among the smokers, the median pack-years was 10.0 pack-years. The majority of participants rated their alcohol consumption as moderate, and the daily number of greens in diet equalled 1–3 portions. The activity and exercise reported by the study population favoured inactivity and not exercising according to recommendations.

**Table 3:** Comparison of exposure patterns and lifestyle habits among cancer types according to Chi-square analysis with Pearson correlation

	**All**	**Breast Cancer**	**Lung Cancer**	**GI cancer**	**Prostate Cancer**	**Other Cancers**	***P*-value**
	N (%)	N (%)	N (%)	N (%)	N (%)	N (%)	
**Smoking Status**							*P* < 0.001
Never-smoker	183 (46.2%)	50 (58.1%)	8 (11.9%)	34 (50.0%)	37 (54.5%)	54 (50.5%)	
Ex-smoker	177 (44.7%)	34 (39.5%)	44 (65.7%)	31 (45.6%)	21 (30.9%)	47 (43.9%)	
Current smoker	36 (9.1%)	2 (2.3%)	15 (22.4%)	3 (4.4%)	10 (14.7%)	6 (5.6%)	
**Pack-years among smokers (Median/Q1, Q3)**	10.0/5.0, 20.0	5.0/2.0, 9.0	20.0/13.0, 40.0	10.0/10.0, 20.5	10.0/10.0, 20.0	10.0/3.0, 10.0	*P* < 0.001
**Heavy smokers over 15 pack-years**	61 (15.2%)	2 (10.0%)	35 (74.5%)	8 (44.4%)	9 (47.4%)	7 (22.6%)	*P* < 0.001
**Exposure to second-hand smoke**							*P* = 0.008
Yes	90 (22.7%)	17 (19.3%)	26 (39.4%)	15 (22.4%)	15 (21.7%)	17 (16.0%)	
No	306 (77.3%)	71 (80.7%)	40 (60.6%)	52 (77.6%)	54 (78.3%)	89 (84.0%)	
**Exposure to VGDF**							*P* < 0.001
Yes	142 (36.0%)	10 (11.4%)	39 (58.2%)	26 (37.7%)	33 (49.3%)	34 (33.0%)	
No	252 (64.0%)	78 (88.6%)	28 (41.8%)	43 (62.3%)	34 (50.7%)	69 (67.0%)	
**Exposure to asbestos**							*P* < 0.001
Yes	51 (13.4%)	3 (3.5%)	17 (27.9%)	9 (13.2%)	14 (21.2%)	8 (7.8%)	
No	331 (86.6%)	82 (96.5%)	44 (72.1%)	59 (86.8%)	52 (78.8%)	94 (92.2%)	
**Alcohol habits**							*P* = 0.381
Does not consume	171 (44.3%)	39 (45.3%)	28 (44.4%)	36 (52.9%)	23 (34.8%)	45 (43.7%)	
Moderate consumption	206 (53.4%)	46 (53.5%)	32 (50.8%)	31 (45.6%)	42 (63.6%)	55 (53.4%)	
Excessive consumption	5 (1.3%)	0 (0.0%)	2 (3.2%)	0 (0.0%)	0 (0.0%)	3 (2.9%)	
Has caused problems	4 (1.0%)	1 (1.2%)	1 (1.6%)	1 (1.5%)	1 (1.5%)	0 (0.0%)	
**Daily portions of greens in diet**							*P* = 0.040
1–3	258 (68.1%)	49 (57.0%)	50 (80.6%)	45 (68.2%)	42 (65.6%)	72 (71.3%)	
4–6	121 (31.9%)	37 (43.0%)	12 (19.4%)	21 (31.8%)	22 (34.4%)	29 (28.7%)	
**Activity per day in hours**							*P* = 0.013
1–2	201 (53.9%)	42 (50.0%)	23 (37.7%)	44 (65.7%)	33 (51.6%)	59 (60.8%)	
3–6	172 (46.1%)	42 (50.0%)	38 (62.3%)	23 (34.3%)	31 (48.4%)	38 (39.2%)	
**Exercise according to recommendations**							*P* = 0.010
Yes	168 (43.9%)	50 (58.8%)	22 (34.9%)	23 (33.8%)	31 (47.7%)	42 (41.2%)	
No	215 (56.1%)	35 (41.2%)	41 (65.1%)	45 (66.2%)	34 (52.3%)	60 (58.8%)	
**Disadvantageous lifestyle habits**							*P* = 0.101
0–1	45 (13.2%)	15 (20.3%)	3 (5.2%)	12 (19.0%)	6 (10.5%)	0 (10.0%)	
2–3	209 (61.1%)	41 (55.4%)	43 (74.1%)	38 (60.3%)	35 (61.4%)	52 (57.8%)	
4–5	88 (25.7%)	18 (24.3%)	12 (20.7%)	13 (20.6%)	16 (28.1%)	29 (32.2%)	

*GI* Gastrointestinal, *Q1* Lower quartile, *Q3* Upper quartile, *VGDF* Vapours, gas, dust and fumes

### Cancer types and socioeconomic status

Comparison of the different cancer types indicated that there were differences in socioeconomic patterns (Table 1). Prostate cancer patients were the oldest, and breast cancer patients were the youngest. Finnish was reported as the mother tongue of the majority of patients in each category, but lung cancer had a shift towards participants who were primarily Finnish speaking (82.1%). Lung cancer patients had the highest proportion of low education (40.3%). In contrast, breast cancer participants had the highest reported educational level. Income was not significantly associated with the cancer types. However, lung cancer patients had the highest proportion and breast cancer patients had the lowest proportion of participants who reported income classified as low income. There was no significant difference whether participants could afford a sudden payment among the cancer types. A common factor for the cancer patients was that the majority of patients had a low occupational level regardless of cancer type. Almost one-third of the patients had compulsory education as the highest attained educational level and the second lowest occupational skill level.

### Cancer types and lifestyle

Table 2 shows the lifestyle patterns reported for the different cancer types in the study population. Regarding smoking habits, current smokers were most abundant in lung cancer (22.4%) and least abundant in breast cancer (2.3%). Lung cancer patients had the highest proportion of heavy smokers with pack-years 15 or above (74.5%), while breast cancer patients had the lowest proportion of heavy smokers (10.0%). Alcohol consumption was not significantly related to the cancer types. The average consumption of greens in diets was low for every cancer type, but lung cancer patients had the highest proportion recorded (80.6%). Inactivity was mostly reported by GI cancer patients (65.7%), and lung cancer patients were the most active group (37.7%). Not exercising according to recommendations was reported the most by GI cancer patients (66.2%) followed by lung cancer (65.1%), and it was reported the least by breast cancer patients (41.2%).

## Discussion

This study sought to explore differences in both SES and lifestyle among cancer patients in Ostrobothnia, Finland. The patient demographics varied significantly among cancer types with different socioeconomic and lifestyle patterns. Lung cancer participants had the lowest SES and the most disadvantageous lifestyle habits, but the contrary was observed in breast cancer participants.

Ross and Wu [21] concluded that high educational level improves health indirectly via favourable occupational and economic situation, suggesting that education may be related to a more favourable occupation and economic situation. With higher education and occupational levels, income increases, contributing to a more favourable economic situation [21]. Therefore, high SES may contribute to a favourable outcome regarding cancer characteristics and present distant metastases. Thus, low SES may contribute to cancer health inequity due to different overlapping factors. In addition to education being related with socioeconomic status, recent interest on its relationship with health literacy has emerged. Stormacq et al. [22] concluded that education was an important determinant of low health literacy levels. Health literacy in turn, was proposed to mediate the relationship between socioeconomic status and health status.

## Socioeconomic status and cancer

Of the major cancer types identified in the present study, lung cancer had the lowest SES in the form of attained educational level. According to Pizzato et al. [23], low SES in lung cancer patients has been observed in other Nordic countries regardless of histological subtype, supporting that low SES is related to lung cancer. Interestingly, in Nordic countries smoking has shifted towards being more common in both men and women with low educational level. Menvielle et al. [24] discussed the cause to be Nordic countries reaching the final stage of the smoking epidemic, resulting in low SES being associated with lung cancer increasingly, regardless of gender. As early as 1953, Doll et al. [6] demonstrated the causality between smoking and lung cancer. Although important, the high proportion of lung cancer among patients with low SES is likely not explained by smoking habits alone [7]. Neuberger and Field [25] found evidence that asbestos, second-hand smoke and occupational exposures are associated with an elevated risk for lung cancer among never-smokers, but their study was limited to the potential of residual confounding effects of smoking. Also, Kreuzer et al. [26] concluded that having worked in an occupation with lung carcinogens is associated with a two-fold increased lung cancer risk. Pukkala et al. [27] found that second-hand smoke and occupational exposures are related to the highest risk of lung cancer regardless of histological subtype.

Breast cancer had the highest SES in the form of attained educational level. In addition, breast cancer had the lowest proportion of metastatic cancer. Although the low proportion of distant metastases in breast cancer may be attributed to breast cancer screening in Finland, there is potential in preventing more cases by optimising screening coverage [28]. Participation in breast cancer screening is also related to SES. Individuals with low SES are less adherent to screening compared to individuals with high SES [29]. This difference was identified as early as 2005 and remains as of today [30]. Screening coverage could be increased by addressing people with lower SES in directed recruitment programmes.

Prostate cancer patients had the second lowest educational level but the second lowest proportion of low income and the lowest proportion of the inability to afford a payment of €1200. This result is likely explained by prostate cancer patients being a heterogeneous group regarding cancer characteristics and prognosis [31]. As early as 1986, Pukkala and Teppo [32] concluded that colorectal cancers are associated with higher SES and education compared to oesophageal and gastric cancers, which are associated with lower SES. Savijärvi et al. [33] confirmed these results in 2019 but reported a shift among men with low education having an increased proportion of colorectal cancer. This change over time may contribute to the GI cancer patient group having the second lowest observed SES as the previously dominant high SES category of colorectal cancer was increasingly associated with low SES. Previously, melanoma has been associated with increased incidence among individuals with high SES, but lower SES is associated with an increased risk of advanced disease at diagnosis [34]. In contrast, neuroendocrine tumours are not associated with low SES or having a metastatic disease at the time of diagnosis [35]. Glioblastoma has been previously reported to be related with higher SES [36], but further studies are needed to define a possible mechanism between this relationship.

## Lifestyle and cancer

Lifestyle habits may contribute to both the development of cancer and treatment success. Regarding a possible dietary effect related to cancer, Mentella et al. [37] found that the Mediterranean diet has a protective effect against cancer onset. In addition, Klement et al. [38] concluded that diet has a possible favourable effect on cancer therapy through effects on gut microbiota. In addition to dietary habits, not exercising according to recommendations and inactivity were related to major cancer types in the present study population with lung cancer patients exercising the least and being the least inactive. Idorn and Straten [39] found evidence supporting that exercise has an effect beyond being healthy and may be therapeutic and improve response to immunotherapy, in animal models. Brown et al. [40] found that there may be a relationship between physical activity and risk reduction in lung cancer. This relationship may be related to the quantity of activity and cancer risk. Confirming results were reported in a study from 2016. Physical activity reduced the risks of several cancer types, including lung cancer [41]. The risk remained after adjusting for confounders, including education. In addition to exercise and diet having a direct effect on cancer risk, they are also related to obesity [42]. Obesity, in turn, is regarded to be associated with increased cancer risk due to hyperinsulinaemia [43]. Previous studies have found a likely relationship with alcohol consumption and cancer pathogenesis. Rumgay et al. [44] found evidence that alcohol may contribute to the burden of cancer via direct and indirect DNA damage.

## Strengths and limitations

The study is limited to relying on self-reported information. Medical records of patients may be lacking on patient-related factors depending on the charting of medical personnel. There is a paucity of research regarding comparison of both SES and lifestyle among cancer types. Any cancer patient was eligible for the study and a certain selection bias may have existed in the form of patients with an active treatment visiting the cancer clinic. Further, there was a random effect based on the selection of patients due to variation in first contact and thus inclusion in the study.

## Clinical impact

There was a need of support in all cancer types, most need in lung cancer and least in breast cancer. Economical support might need to be offered to one out of three cancer patients. Self-cost for hospital stays, travel, and medicines exceeds €1200, limit considered as absolute poverty in Finland. Mackenbach et al. [45] found that mortality has decreased in all of Europe among those with low education however, the relative index of inequality has increased over all for both men and women, in Finland. One out of four cancer patients lived alone and might benefit from a support network. Patients with poor dietary habits may benefit from nutritional counselling regardless of disease stage and choice of treatment. Most cancer patients would benefit from exercise to improve performance status. Support for stopping smoking and substance abuse should be readily available. Thankfully, stakeholders such as local cancer organization offers support to patients when public health care is limited. Finland participates in the Joint Action Health Equity Europe project, involving ministries of health, addressing inequalities in the population [46].

## Conclusions

The community-based study indicated significant socioeconomic and lifestyle differences among cancer types in Ostrobothnia. Vulnerable patients found in all cancer types may benefit from extra resources to alleviate disparities in cancer care and as a part of this community-based study workshop between local health care providers and patient organizations addressed the needs of cancer patients. As an example, to identify disadvantaged patients, comprehensive baseline survey is planned to be incorporated in clinical practice to be completed by all new patients. Depending on the response, health care providers could optimise care through i.e. referral to nutritional counselling. Those needing social and financial support may benefit of contact with a local cancer organization. Local cancer organization offers support person to help with navigation in cancer care and this may benefit most those with low education or low health literacy to make lifestyle changes needed for better cancer survival.

## Data Availability

Due to its proprietary nature and Finnish General Data Protection regulation data cannot be made public, request should be addressed to Heidi Andersén.
